# Relationship between albumin-corrected anion gap and lumbar spine bone mineral density: a cross-sectional study

**DOI:** 10.3389/fragi.2025.1511294

**Published:** 2025-02-11

**Authors:** Aiguo Liu, Ting Ying, Shuang Deng, Chenxu Wang, Ziwen Zhao, Sitong Zhang, Han Xiao, Chengqing Yi, Dejian Li

**Affiliations:** ^1^ Department of Orthopedics, The First Affiliated Hospital of Henan University, Kaifeng, China; ^2^ Department of Orthopedics, Shanghai Pudong Hospital, Fudan University Pudong Medical Center, Shanghai, China

**Keywords:** ACAG, BMD, osteoporosis, NHANES, cross-sectional analysis

## Abstract

**Objectives:**

This study aimed to investigate the relationship between albumin-corrected anion gap (ACAG) and lumbar spine bone mineral density (BMD) in a diverse population, assessing how variations in ACAG levels correlate with changes in lumbar spine BMD and the potential implications for osteoporosis risk.

**Methods:**

A cross-sectional analysis was conducted involving 3,057 participants (1,555 males and 1,502 females). Participants were stratified into quartiles based on baseline ACAG levels. Demographic and clinical characteristics were analyzed, including age, sex, education level, body mass index (BMI), and prevalence of diabetes and hypertension. The association between ACAG and lumbar spine BMD was evaluated using multiple regression models, and a generalized additive model was employed to identify potential nonlinear relationships.

**Results:**

The analysis revealed a significant negative correlation between ACAG and lumbar spine BMD (*P* < 0.001). For each 1-unit increase in ACAG, BMD decreased with β coefficients of −0.004 to −0.005 across various models. Quartile analysis indicated that participants in the highest ACAG quartile (≥19.55) experienced the most substantial reductions in BMD (β coefficients ranging from −0.034 to −0.036, *P* < 0.001). Furthermore, a U-shaped relationship was identified, with a turning point at an ACAG value of 22.15, indicating that lower ACAG levels were associated with decreased BMD, while higher levels showed a positive effect. Subgroup analyses by sex demonstrated consistent findings, with significant associations in both males and females.

**Conclusion:**

The findings underscore a significant association between elevated ACAG levels and reduced lumbar spine BMD, suggesting that ACAG may serve as a valuable biomarker for assessing osteoporosis risk. The identified nonlinear relationship further emphasizes the complexity of metabolic influences on bone health. These results warrant further investigation into the mechanisms underlying ACAG’s impact on bone density and its potential role in osteoporosis prevention strategies.

## Introduction

Osteoporosis is a widespread chronic condition marked by a decline in bone mineral density (BMD) and the degradation of bone microarchitecture. This disorder considerably affects the health of middle-aged and elderly populations worldwide. The international osteoporosis foundation estimates that roughly one-third of women and one-fifth of men over the age of 50 are at risk of developing osteoporosis or osteopenia (Cha et al., 2023). This prevalence is increasing annually due to the aging global population (Yoo and Lee, 2021). The etiology of osteoporosis is multifaceted, shaped by an intricate interplay of genetic predispositions and environmental influences. The principal risk factors include non-modifiable elements such as age, sex, and genetic predisposition, as well as modifiable lifestyle factors (Ji et al., 2022; Wang et al., 2023a). Ensuring sufficient consumption of essential nutrients, particularly calcium and vitamin D, is paramount for the maintenance of optimal skeletal health (Marshall et al., 2020; Shangguan et al., 2022). Additionally, recent studies have highlighted the importance of other nutritional elements, emphasizing the need to investigate the impact of various dietary factors on bone metabolism (Chen et al., 2019; Zeng et al., 2021).

The growing recognition of the influence of lifestyle and nutrition has led to efforts to identify novel risk factors and biomarkers for osteoporosis (Fang et al., 2018; Gu et al., 2019). Improved risk assessment methods are essential for the prevention and effective management of this disease, which not only leads to significant morbidity due to osteoporotic fractures but also imposes substantial economic burdens on healthcare systems (Park et al., 2023; Abd Elsalam, 2022). As research progresses, it will be vital to gain a deeper understanding of these complex interactions to develop effective preventive strategies that can mitigate bone loss and enhance the overall quality of life for those at risk (Yao et al., 2020; Khani Jeihooni et al., 2021).

The anion gap (AG) is a crucial parameter for assessing the concentration gradient of cations and anions in the blood. It is a widely utilized tool for evaluating acid-base balance and classifying metabolic acidosis (Cheng et al., 2020). However, albumin molecules carry a net negative charge, which means that changes in their concentration can affect AG outcomes in patients with hypoalbuminaemia. It was therefore proposed that an albumin-corrected anion gap (ACAG) be employed to circumvent the potential for false negative results (Lu et al., 2024). Research has shown that a heightened level of ACAG is correlated with an increased mortality rate in hospitalized patients suffering from acute pancreatitis (Li et al., 2023). In recent years, research into the relationship between ACAG and osteoporosis has attracted growing interest. Osteoporosis is a complex chronic disease that is typically associated with electrolyte imbalance. There is a close relationship between AG and acid-base balance in the body, which may affect bone metabolism. It may therefore be surmised that ACAG, as a potential biomarker, may be associated with changes in bone density and the risk of osteoporosis. Nevertheless, the existing literature on the relationship between ACAG and osteoporosis remains relatively limited, and further research is required to elucidate the underlying interactions and mechanisms.

Therefore, a cross-sectional study was conducted based on the National Health and Nutrition Examination Survey (NHANES) (2017–2018) with the objective of determining the correlation between ACAG and Lumbar Spine BMD. Furthermore, we sought to ascertain whether sex influenced the correlation between ACAG and lumbar spine BMD. The objective was to explore the linear or nonlinear relationship between ACAG and lumbar spine BMD and to investigate whether ACAG levels have potential value in predicting the risk of osteoporosis or bone loss.

## Methods

### Study design and population

The data employed in this study were derived from NHANES, spanning the period from 2017 to 2018. The NHANES (https://www.cdc.gov/nchs/nhanes/index.htm) is affiliated with the Centers for Disease Control and Prevention (CDC) in the United States. Its purpose is to assess the health and nutritional status of American residents, with updates occurring every 2 years. The NHANES research protocol has been approved by the Ethics Review Committee of the National Health Statistics Research Center, and each participant has provided written informed consent. The NHANES website (https://wwwn.cdc.gov/nchs/nhanes/ContinuousNhanes/Documents.aspx?BeginYear=2017) offers comprehensive guidance on ethical considerations and the process of written informed consent.

**FIGURE 1:**
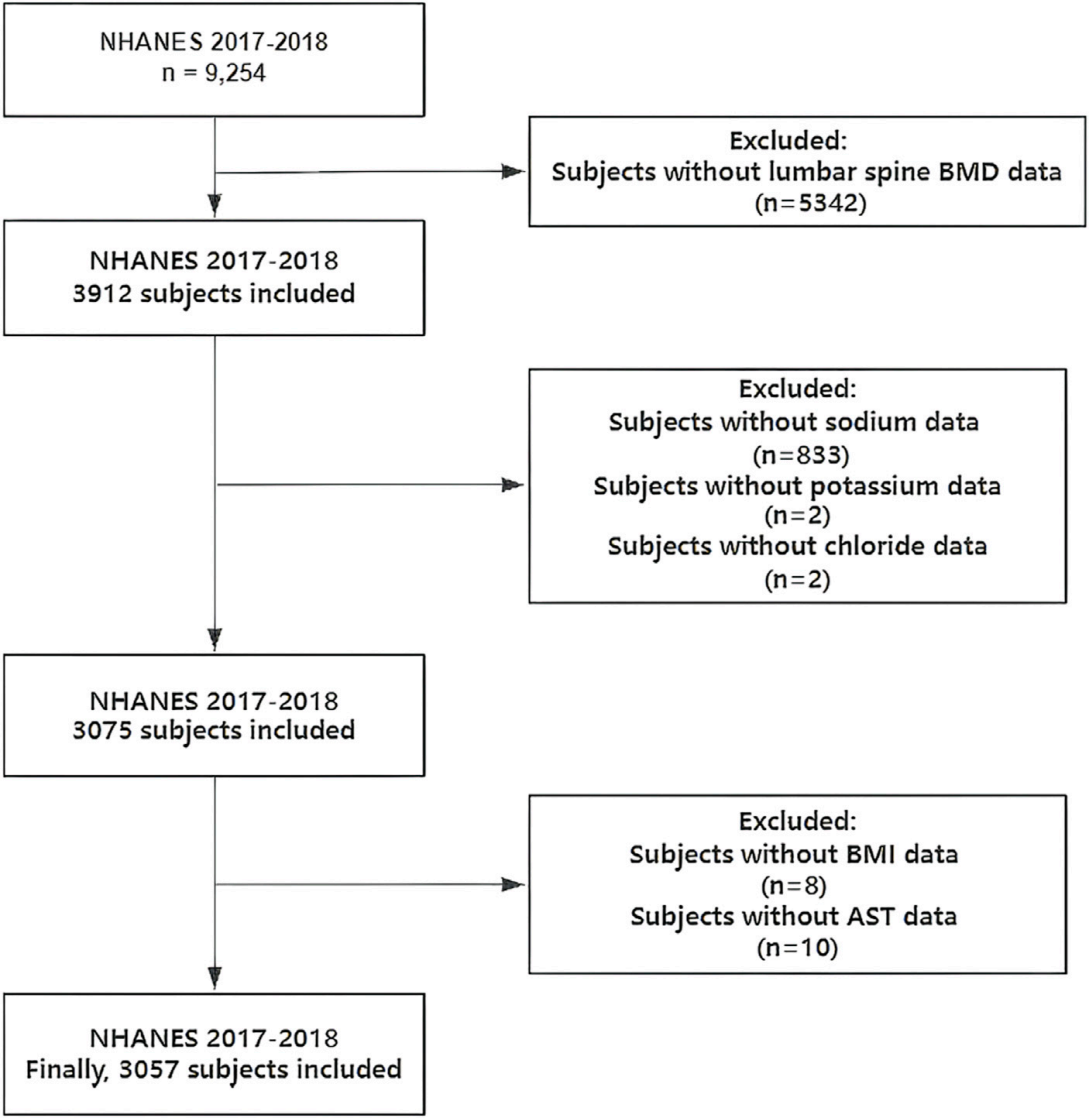
Flowchart of participants selection. NHANES, National Health and Nutrition Examination Survey; BMD, bone mineral density; BMI, body mass index; AST, aspartate aminotransferase.

A total of 9,254 samples were included. Exclusion criteria: missing lumbar spine BMD data (n = 5,342); missing sodium data (n = 833); missing potassium data (n = 2); missing chloride data (n = 2); missing body mass index (BMI) data (n = 8); missing aspartate aminotransferase (AST) data (n = 10). Finally, we enrolled 3,057 samples for analysis (Figure 1).

### BMD testing

BMD was evaluated through the application of dual-energy X-ray absorptiometry (DXA) scans. All participants in this study underwent BMD assessment, which was performed by certified radiologic technologists utilizing Hologic QDR-4500 A fan-beam densitometers (Hologic; Bedford, MA). The resulting data were analyzed using Hologic APEX software, version 4.0. Additional information regarding the BMD testing procedure can be found on the NHANES website (https://wwwn.cdc.gov/nchs/nhanes/continuousnhanes/manuals.aspx?Cycle=2017-2018).

### Variable extraction and ACAG calculation

The demographic characteristics of the participants included age, sex, education level, BMI, poverty income ratio (PIR). Smoking status (Never smokers: Those who have not smoked at least 100 cigarettes in their lifetime. Former smokers: Those who have smoked at least 100 cigarettes in their lifetime but do not currently smoke. Current smokers: Those who have smoked at least 100 cigarettes in their lifetime and currently smoke every day or some days.) and alcohol drinking status (Non-consumers: Individuals who have not consumed any type of alcohol in the past 12 months. Consumers: Individuals who have consumed any type of alcohol in the past 12 months) were collected from self-reported questionnaires in the Mobile examination centers. Furthermore, data regarding hypertension and diabetes were also collected via self-report questionnaires. Serum biochemistry profiles were obtained for the following parameters: total calcium, alanine aminotransferase (ALT), AST, 25-hydroxyvitamin D, sodium, potassium, chloride, bicarbonate, and albumin. To mitigate the potential for collinearity, sodium, potassium, chloride, bicarbonate, and albumin were utilized exclusively for the calculation of ACAG and were subsequently excluded from further statistical analyses.

AG was calculated according to the following equation: AG (mmol/L) = (sodium + potassium) - (chloride + bicarbonate). ACAG was determined using the formula: ACAG (mmol/L) = [4.4 - albumin (g/dL)] * 2.5 + AG (Hatherill et al., 2002).

### Statistical analysis

The baseline characteristics of all participants included in the final analysis are presented as either mean values for continuous variables or proportions for categorical variables. The linear relationship between ACAG and lumbar spine BMD was assessed using multivariate linear regression models. Additionally, a subgroup analysis was performed within these models to explore the linear relationship between ACAG and lumbar spine BMD across various populations, stratified by sex. The non-linear relationship was further characterized through the application of smooth curve fittings and generalized additive models. If an inflection point was identified, it was calculated using two-piecewise linear regression models with a recursive algorithm. P-values less than 0.05 were deemed statistically significant. All statistical analyses were conducted using R software (version 4.0.3; https://www.R-project.org) and EmpowerStats (version 6.0; http://www.empowerstats.com).

## Results

### Participant characteristics

There were 3,057 participants, including 1,555 males and 1,502 females. Table 1 presents the demographic and clinical characteristics of patients stratified by quartiles of baseline ACAG (Q1 to Q4). The number of participants in each quartile were 759, 756, 753, and 789, respectively. Although there was no significant difference in age across the groups (*P* = 0.672), the sex distribution revealed a higher proportion of males in the lower ACAG group (Q1: 56.13%), which decreased to 45.37% in the higher ACAG group (Q4, *P* < 0.001). Regarding education level, the percentage of patients with education below high school increased in the higher ACAG group (Q4: 40.68%, *P* = 0.013). BMI analysis showed that the proportion of participants with low BMI (<25 kg/m^2^) decreased from 44.14% in Q1 to 33.46% in Q4 (*P* < 0.001), while the percentage of obese participants (≥30 kg/m^2^) increased from 26.48% in Q1 to 39.29% in Q4 (*P* < 0.001). The prevalence of diabetes and hypertension also significantly increased in the higher ACAG group (diabetes: Q4: 9.25%, *P* < 0.001; hypertension: Q4: 20.66%, *P* = 0.027). Biochemical indicators demonstrated a significant decrease in lumbar spine BMD with increasing ACAG (*P* < 0.001), while electrolyte levels and albumin also exhibited significant differences across quartiles (*P* < 0.001).

**TABLE 1:** The demographic and clinical characteristics of the patients by quartiles of baseline ACAG.

Variable^a^	ACAG quartiles				*P* Value
	Q1(<16.80)	Q2(16.80 to <18.20)	Q3(18.20 to <19.55)	Q4(≥19.55)	
Participants	759	756	753	789	
Age(year)	33.93 ± 13.79	33.63 ± 14.73	33.20 ± 14.96	33.12 ± 15.02	0.672
Sex, n (%)					<0.001
Male	426 (56.13%)	403 (53.31%)	368 (48.87%)	358 (45.37%)	
Female	333 (43.87%)	353 (46.69%)	385 (51.13%)	431 (54.63%)	
Education level, n (%)					0.013
Under high school	252 (33.20%)	291 (38.49%)	298 (39.58%)	321 (40.68%)	
High school or above high school	507 (66.80%)	465 (61.51%)	455 (60.42%)	468 (59.32%)	
PIR n, (%)					0.822
<1	220 (28.99%)	236 (31.22%)	225 (29.88%)	236 (29.91%)	
≥1	539 (71.01%)	520 (68.78%)	528 (70.12%)	553 (70.09%)	
BMI, n (%)					<0.001
<25 kg/m^2^	335 (44.14%)	282 (37.30%)	298 (39.58%)	264 (33.46%)	
25 ≤ BMI <30 kg/m^2^	223 (29.38%)	225 (29.76%)	212 (28.15%)	215 (27.25%)	
≥30 kg/m^2^	201 (26.48%)	249 (32.94%)	243 (32.27%)	310 (39.29%)	
Alcohol drinking status n, (%)					0.657
Yes	67 (8.83%)	73 (9.66%)	81 (10.76%)	77 (9.76%)	
No	692 (91.17%)	683 (90.34%)	672 (89.24%)	712 (90.24%)	
Smoke status, n (%)					0.013
Never	530 (69.83%)	570 (75.40%)	533 (70.78%)	575 (72.88%)	
Current	112 (14.76%)	103 (13.62%)	121 (16.07%)	135 (17.11%)	
Former	117 (15.42%)	83 (10.98%)	99 (13.15%)	79 (10.01%)	
AST(U/L)	21.61 ± 13.27	21.51 ± 11.46	22.09 ± 13.71	22.44 ± 13.02	0.447
ALT(U/L)	21.18 ± 20.72	22.01 ± 17.07	22.68 ± 18.40	23.49 ± 19.55	0.103
Serum total calcium (mmol/L)	2.33 ± 0.09	2.34 ± 0.09	2.34 ± 0.08	2.33 ± 0.10	0.234
Serum 25(OH)D (nmol/L)	59.73 ± 25.05	61.68 ± 26.07	61.53 ± 24.76	59.17 ± 25.61	0.126
lumbar spine BMD (g/cm^2^)	1.05 ± 0.16	1.02 ± 0.15	1.03 ± 0.16	1.01 ± 0.16	<0.001
Sodium (mmol/L)	139.60 ± 2.26	139.79 ± 2.39	140.12 ± 2.53	140.61 ± 2.92	<0.001
Potassium (mmol/L)	4.01 ± 0.32	4.04 ± 0.34	4.06 ± 0.32	4.09 ± 0.34	<0.001
Chloride (mmol/L)	101.39 ± 2.19	101.15 ± 2.34	100.95 ± 2.50	100.64 ± 2.83	<0.001
Bicarbonate (mmol/L)	27.18 ± 2.05	25.74 ± 1.91	24.99 ± 1.97	23.66 ± 2.12	<0.001
Albumin (g/dL)	4.21 ± 0.30	4.17 ± 0.32	4.16 ± 0.32	4.11 ± 0.33	<0.001
Diabetes, n (%)					<0.001
Yes	29 (3.82%)	27 (3.57%)	41 (5.44%)	73 (9.25%)	
No	730 (96.18%)	729 (96.43%)	712 (94.56%)	716 (90.75%)	
Hypertension, n (%)					0.027
Yes	114 (15.02%)	135 (17.86%)	125 (16.60%)	163 (20.66%)	
No	645 (84.98%)	621 (82.14%)	628 (83.40%)	626 (79.34%)	

Abbreviations: ACAG, albumin-corrected anion gap; PIR, poverty income ratio; BMI, body mass index; AST, aspartate aminotransferase; ALT, alanine aminotransferase; BMD, bone mineral density, 25(OH)D 25-hydroxyvitamin D.

^a^ Data are presented as number (%) or mean ± standard deviation.

### Association between ACAG and lumbar spine BMD

Table 2 presents the association between ACAG and lumbar spine BMD. For each 1-unit increase in ACAG, the BMD decreased significantly, with β coefficients of −0.004 (95% CI: −0.007, −0.002) in Model 1, -0.004 (95% CI: −0.006, −0.002) in Model 2, and -0.005 (95% CI: −0.007, −0.002) in Model 3, all indicating a statistically significant association (*P* < 0.001). Quartile analysis revealed that participants in Q2 (16.80 to <18.20) showed a decrease in BMD of −0.022 (95% CI: −0.038, −0.007) in Model 1, -0.020 (95% CI: −0.035, −0.005) in Model 2, and -0.020 (95% CI: −0.035, −0.005) in Model 3 (both *P* < 0.01 and *P* < 0.05). Similarly, Q3 (18.20 to <19.55) showed decreases of −0.021 (95% CI: −0.036, −0.005) in Model 1, -0.018 (95% CI: −0.034, −0.003) in Model 2, and -0.019 (95% CI: −0.034, −0.004) in Model 3 (both *P* < 0.01 and *P* < 0.05). In Q4 (≥19.55), the reductions in BMD were even greater, with values of −0.034 (95% CI: −0.050, −0.019) in Model 1, -0.032 (95% CI: −0.047, −0.017) in Model 2, and -0.036 (95% CI: −0.051, −0.021) in Model 3 (all *P* < 0.001). The trend analysis confirmed a significant association across all models (*P for trend* < 0.0001).

**TABLE 2:** Association between ACAG and lumbar spine BMD.

ACAG	β (95% CI)		
	Model 1	Model 2	Model 3
Per 1 increment	−0.004 (−0.007, −0.002)***	−0.004 (−0.006, −0.002)**	−0.005 (−0.007, −0.002)***
Quartile			
Q1(<16.80)	Reference	Reference	Reference
Q2(16.80 to <18.20)	−0.022 (−0.038, −0.007)**	−0.020 (−0.035, −0.005)*	−0.020 (−0.035, −0.005)*
Q3(18.20 to <19.55)	−0.021 (−0.036, −0.005)**	−0.018 (−0.034, −0.003)*	−0.019 (−0.034, −0.004)*
Q4(≥19.55)	−0.034 (−0.050, −0.019)***	−0.032 (−0.047, −0.017)***	−0.036 (−0.051, −0.021)***
*P for trend*	<0.0001	<0.0001	<0.0001

Abbreviations: ACAG albumin-corrected anion gap, PIR poverty income ratio,BMI body mass index, AST aspartate aminotransferase, ALT alanine aminotransferase, BMD bone mineral density, 25(OH)D 25-hydroxyvitamin D.

*P < 0.05.

**P < 0.01.

***P < 0.001.

Model 1 was adjusted for none.

Model 2 was adjusted for Age,Sex, Education level, PIR.

Model 3 was adjusted for Age, Sex, Education level, PIR, Alcohol drinking status, Smoke status, Diabetes, Hypertension, BMI, AST, ALT, Serum total calcium, Serum 25(OH)D.

### Identification of nonlinear relationship between ACAG and lumbar spine BMD

**FIGURE 2:**
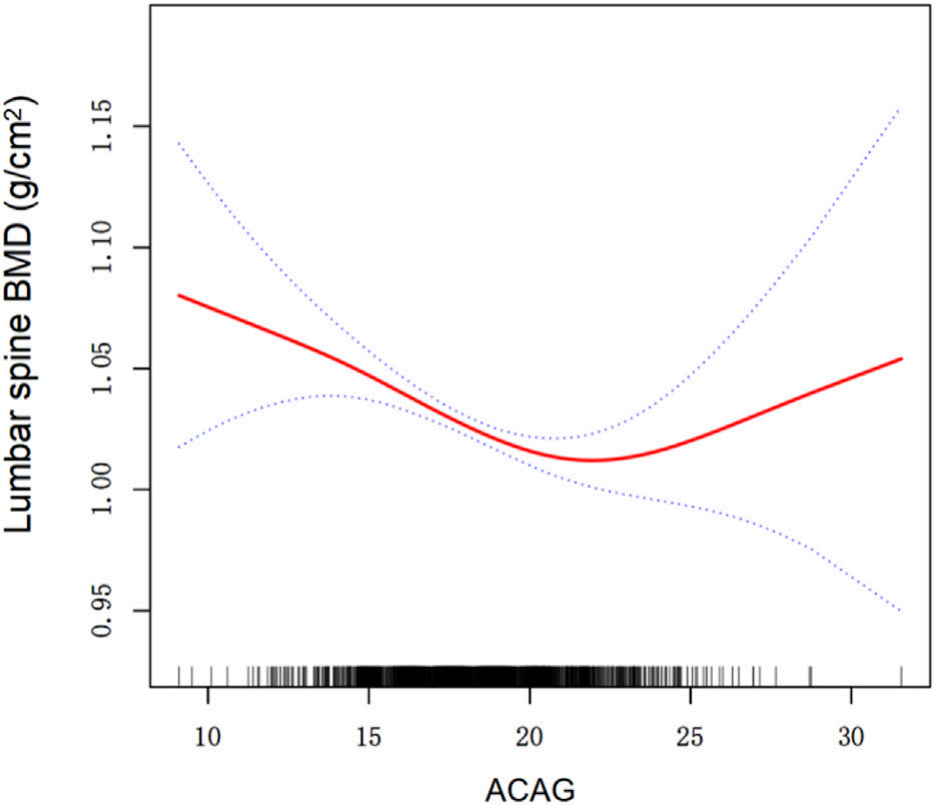
Association between ACAG and lumbar spine BMD. A threshold, nonlinear association between ACAG and lumbar spine BMD was found in a generalized additive model (GAM). The solid red line represents the smooth curve fit between variables. Dashed line represent the 95% of confidence interval from the fit. All adjusted for Age, Sex, Education level, PIR, Alcohol drinking status, Smoke status, Diabetes, Hypertension, BMI, AST, ALT, Serum total calcium, Serum 25(OH)D. Abbreviations: ACAG albumin-corrected anion gap, PIR poverty income ratio,BMI body mass index, AST aspartate aminotransferase, ALT alanine aminotransferase, BMD bone mineral density, 25(OH)D 25-hydroxyvitamin D.

Using the generalized additive model, a U-shaped association between ACAG and lumbar spine BMD was detected (Table 3; Figure 2). Table 3 presents the results of the threshold effect analysis of ACAG on lumbar spine BMD. In Model I, the linear effect of ACAG on BMD was found to be statistically significant, with a β coefficient of −0.005 (95% CI: −0.007, −0.002) and a *P* value of 0.0002. Model II identified a turning point (K) at 22.15. For values of ACAG below the turning point, the association with BMD was negative, with a β coefficient of −0.006 (95% CI: −0.009, −0.004) and a highly significant *P* value of <0.0001. Conversely, for values of ACAG equal to or greater than the turning point, the effect on BMD was positive, indicated by a β coefficient of 0.013 (95% CI: 0.001, 0.026) and a *P* value of 0.0417. The likelihood ratio test (LRT) yielded a *P* value of 0.005, indicating that the threshold effect is statistically significant. The 95% confidence interval for the turning point was estimated to be 20.5 to 22.25.

**TABLE 3:** Threshold effect analysis of ACAG and lumbar spine BMD.

Model	β (95% CI)	*P* Value
Model Ⅰ		
One line effect	−0.005 (−0.007, −0.002)	0.0002
Model Ⅱ		
Turning point (K)	22.15	
ACAG < K	−0.006 (−0.009, −0.004)	<0.0001
ACAG ≥ K	0.013 (0.001, 0.026)	0.0417
*P* value for LRT test*	0.005	
95% Cl for Turning point	20.5, 22.25	

Data were presented as β (95% CI) *P* value; ModelⅠ,linear analysis;ModelⅡ,non-linear analysis.

Adjusted for Age, Sex, Education level, PIR, alcohol drinking status, Smoke status, Diabetes, Hypertension, BMI, AST, ALT, serum total calcium, Serum 25(OH)D.

*P < 0.05 indicates that ModelⅡ is significantly different from ModelⅠ.

Abbreviations: ACAG, albumin-corrected anion gap; PIR, poverty income ratio; BMI, body mass index; AST, aspartate aminotransferase; ALT, alanine aminotransferase; BMD, bone mineral density, 25(OH)D 25-hydroxyvitamin D, cl confidence interval, LRT, logarithm likelihood ratio test.

### Association between ACAG and lumbar spine BMD by sex

The subgroup analysis stratified by sex is shown in Table 4. Table 4 illustrates the association between ACAG and lumbar spine BMD in different models for both males and females. In males, the per 1 increment of ACAG was associated with a significant decrease in BMD across all models, with β coefficients of −0.005 (95% CI: −0.008, −0.001) in Model 1, -0.004 (95% CI: −0.008, −0.001) in Model 2, and -0.005 (95% CI: −0.008, −0.001) in Model 3. For the quartile analysis, while Q2 and Q3 did not show significant associations, Q4 (≥19.55) reflected a notable decrease in BMD, with β coefficients of −0.033 (95% CI: −0.056, −0.010), −0.030 (95% CI: −0.053, −0.007), and −0.032 (95% CI: −0.055, −0.009) across the models. The *P* values for trend in males were 0.006, <0.0001, and <0.0001 for Models 1, 2, and 3, respectively.

**TABLE 4:** Association between ACAG and lumbar spine BMD in diferent models among male and female.

ACAG	β (95% CI)		
	Model 1	Model 2	Model 3
Male			
Per 1 increment	−0.005 (−0.008, −0.001)*	−0.004 (−0.008, −0.001)*	−0.005 (−0.008, −0.001)*
Q1(<16.80)	Reference	Reference	Reference
Q2(16.80 to <18.20)	−0.019 (−0.041, 0.004)	−0.016 (−0.038, 0.006)	−0.015 (−0.037, 0.007)
Q3(18.20 to <19.55)	−0.023 (−0.046, 0.000)	−0.020 (−0.042, 0.003)	−0.019 (−0.042, 0.004)
Q4(≥19.55)	−0.033 (−0.056, −0.010)**	−0.030 (−0.053, −0.007)**	−0.032 (−0.055, −0.009)**
*P for trend*	0.006	<0.0001	<0.0001
Female			
Per 1 increment	−0.005 (−0.008, −0.001)**	−0.004 (−0.007, −0.001)*	−0.005 (−0.008, −0.002)**
Q1(<16.80)	Reference	Reference	Reference
Q2(16.80 to <18.20)	−0.027 (−0.048, −0.005)*	−0.026 (−0.047, −0.004)*	−0.026 (−0.047, −0.005)**
Q3(18.20 to <19.55)	−0.021 (−0.042, −0.000)**	−0.019 (−0.040, 0.002)	−0.020 (−0.040, 0.001)
Q4(≥19.55)	−0.039 (−0.060, −0.019)***	−0.037 (−0.057, −0.017)***	−0.042 (−0.062, −0.022)***
*P for trend*	<0.0001	0.002	<0.0001

Abbreviations: ACAG albumin-corrected anion gap, PIR poverty income ratio,BMI body mass index, AST aspartate aminotransferase, ALT alanine aminotransferase, BMD bone mineral density, 25(OH)D 25-hydroxyvitamin D.

*P < 0.05.

**P < 0.01.

***P < 0.001.

Model 1 was adjusted for none.

Model 2 was adjusted for Age, Education level, PIR.

Model 3 was adjusted for Age, Education level, PIR, Alcohol drinking status, Smoke status, Diabetes, Hypertension, BMI, AST, ALT, Serum total calcium, Serum 25(OH)D.

In females, the results were similarly significant, with the per 1 increment of ACAG showing β coefficients of −0.005 (95% CI: −0.008, −0.001) in Model 1, -0.004 (95% CI: −0.007, −0.001) in Model 2, and -0.005 (95% CI: −0.008, −0.002) in Model 3. Notably, in the quartile analysis, Q2 (16.80 to <18.20) exhibited a significant decrease in BMD with β coefficients of −0.027 (95% CI: −0.048, −0.005), −0.026 (95% CI: −0.047, −0.004), and −0.026 (95% CI: −0.047, −0.005). Q4 also showed substantial reductions with β coefficients of −0.039 (95% CI: −0.060, −0.019), −0.037 (95% CI: −0.057, −0.017), and −0.042 (95% CI: −0.062, −0.022). The *P* values for trend in females were all significant (<0.0001, 0.002, <0.0001) across the models.

## Discussion

In our investigation of the association between ACAG and lumbar spine BMD, we identified a significant inverse relationship that persisted across multiple statistical models and analyses. Participants in the highest quartile of ACAG (Q4, ≥19.55) exhibited a markedly lower lumbar spine BMD of 1.01 ± 0.16 g/cm^2^ compared to those in the lowest quartile (Q1, <16.80) with a BMD of 1.05 ± 0.16 g/cm^2^ (*P* < 0.001). Multivariable regression analysis revealed that for each unit increase in ACAG, there was a decrease in BMD of −0.005 g/cm^2^ (95% CI: −0.007, −0.002) across all models, reinforcing the robustness of this association. This negative trend in BMD persisted irrespective of participant characteristics such as age, sex, education, and comorbidities like diabetes and hypertension. Notably, the threshold effect analysis indicated a turning point at ACAG levels of 22.15, suggesting that higher levels of ACAG may contribute to an increase in BMD deterioration. These findings underscore the potential role of ACAG as a biomarker for reduced bone density, indicating that elevated ACAG may adversely affect lumbar spine health. Thus, monitoring ACAG levels may be crucial in assessing and managing bone health, particularly in populations at risk for osteoporosis.

Sodium, potassium, chloride, and bicarbonate are essential electrolytes that significantly influence bone density and the risk of osteoporosis. Recent studies have established a clear association between high sodium intake and reduced bone density, primarily attributed to increased urinary calcium excretion resulting from excessive sodium consumption. This phenomenon disrupts calcium homeostasis, leading to impaired bone mineralization and an elevated risk of osteoporosis (Takase et al., 2023; Saad et al., 2020). For instance, a study highlighted that excessive salt intake correlates with decreased bone density in the general female population, particularly among older adults, indicating that sodium’s impact on bone health is exacerbated with age (Takase et al., 2023). Furthermore, high dietary sodium has been linked to enhanced bone resorption and diminished bone formation, which collectively contribute to a decline in BMD (Saad et al., 2020; Dar et al., 2018).

In contrast, potassium intake has been associated with protective effects on bone health. Potassium plays a crucial role in regulating acid-base balance, which can mitigate the acid load induced by certain diets. This regulation is vital for maintaining bone density, as it helps to reduce the negative effects of dietary-induced acidosis on bone health (Fasihi et al., 2021). Research has shown that higher potassium intake can lead to improved calcium retention and a reduction in urinary calcium excretion, thereby supporting bone mineralization and density (Nazarena, 2021).

Chloride, as a co-anion of sodium, also influences bone health through its role in modulating acid-base balance. The interaction between sodium and chloride can affect systemic pH levels, which in turn impacts bone metabolism (Takase et al., 2023). Bicarbonate is particularly important for maintaining systemic pH levels; adequate bicarbonate levels can alleviate the adverse effects of acid load on bone. Studies indicate that bicarbonate supplementation can enhance calcium retention and improve bone matrix quality, potentially leading to increased bone density (Marino et al., 2023). For example, bicarbonate supplementation has been shown to favorably affect bone resorption and calcium excretion in older adults, suggesting its role in promoting bone health (Yamauchi et al., 2023).

Given the intricate interactions among these electrolytes, understanding their combined effects is essential for developing effective strategies for the prevention and treatment of osteoporosis. Dietary interventions that consider the balance of sodium, potassium, chloride, and bicarbonate intake may prove beneficial in maintaining bone health and preventing osteoporosis in various populations.

AG has emerged as a potential biomarker for assessing bone density and the risk of osteoporosis. Elevated AG levels may indicate an increased acid load in the body, leading to a state of metabolic acidosis. This condition has been linked to enhanced bone resorption and decreased bone formation due to the mobilization of calcium from bone tissue to buffer excess acidity (Cheng et al., 2020). Specifically, metabolic acidosis can stimulate osteoclast activity, which is responsible for bone resorption, while simultaneously impairing osteoblast function, which is crucial for bone formation (Altınsoy and Unat, 2024; Rudkin et al., 2021). Several studies have demonstrated a negative correlation between higher AG and BMD, suggesting that individuals with elevated AG are at an increased risk for osteoporosis (Duyan et al., 2023).

The underlying mechanisms may involve alterations in calcium and phosphate metabolism. Acidic conditions promote urinary calcium excretion while impairing intestinal calcium absorption, leading to a net loss of calcium that is detrimental to bone health (Rudkin et al., 2022; Zhang et al., 2020). Furthermore, chronic metabolic acidosis can trigger the release of parathyroid hormone (PTH), which further exacerbates bone loss by stimulating osteoclast activity and increasing calcium mobilization from the bone (Marini et al., 2020; Wang et al., 2020). For instance, a study indicated that elevated AG levels were associated with increased PTH levels, which correlated with reduced BMD in postmenopausal women (Ahmad et al., 2020; Zheng et al., 2022).

ACAG has emerged as a significant biomarker for the assessment of bone mineral density and the risk of osteoporosis. Recent studies have demonstrated that elevated ACAG levels may serve as a indicator of underlying metabolic disorders, which are strongly associated with increased bone resorption. Elevated ACAG values are generally indicative of electrolyte imbalance, particularly in cases of hypoalbuminemia, where changes in the anion gap due to low albumin levels may affect calcium homeostasis and consequently impact bone health (Hu et al., 2023). Specifically, hypoalbuminemia leads to a decrease in total protein levels, resulting in an inaccurate calculation of the anion gap. This discrepancy can potentially obscure the true status of bone mineral density changes, leading to an inaccurate assessment of osteoporosis risk, particularly in the elderly population (Lambert and Abramowitz, 2021). Studies have demonstrated a direct correlation between low albumin levels and reduced bone mineral density, emphasising the pivotal role of albumin in regulating bone metabolism (Gao et al., 2023). Concurrently, elevated ACAG levels can precipitate a metabolic acidosis state, thereby stimulating PTH secretion. Research has demonstrated that heightened PTH concentrations promote bone mineral demineralisation and escalate the risk of osteoporosis (Wang et al., 2023b). Furthermore, the increased ACAG-induced acidic environment has been demonstrated to inhibit the function of osteoblasts whilst concomitantly promoting the activity of osteoclasts, thus giving rise to diminished bone formation and augmented bone resorption, a phenomenon that is especially pronounced in the elderly population (Jian et al., 2023). It is imperative to monitor ACAG levels, as they offer invaluable insights into bone health and facilitate the elucidation of the pathophysiology underlying osteoporosis. A comprehensive understanding of the relationship between ACAG and BMD, particularly in patients with hypoalbuminemia, has the potential to inform strategies for the prevention and management of osteoporosis in high-risk populations. In this regard, further research investigating ACAG in diverse populations is warranted to fully elucidate its potential as a predictive marker for osteoporosis and to optimise clinical interventions.

It should be noted that this study is not without limitations. Firstly, it should be noted that this is a cross-sectional study, which has certain limitations in terms of its design and scope in comparison to cohort studies. Secondly, the inclusion of only those individuals with complete data and the exclusion of subjects with missing data may result in a degree of bias. Thirdly, the study is unable to explore etiological hypotheses and therefore cannot determine the causal relationship between ACAG and lumbar spine BMD. Fourthly, the participants included in the final analysis are based on the general population of the United States. It should be noted that this approach may not be extrapolated to other countries, given the potential for differences in genetic, linguistic, cultural, and environmental factors among different countries and regions.

In summary, our research findings indicate a negative correlation between ACAG and Lumbar Spine BMD, particularly in females. ACAG may be a potential reliable biomarker for predicting lumbar spine BMD, therefore, monitoring ACAG levels may be necessary in assessing and managing lumbar spine bone health.

## Data Availability

The original contributions presented in the study are included in the article/supplementary material, further inquiries can be directed to the corresponding authors.
